# Herbicidal fungal strain isolated from soil in Xinjiang, China

**DOI:** 10.1128/spectrum.01589-24

**Published:** 2024-10-17

**Authors:** Yanhong Tang, Wei Chen, Fengting He, Tongyi Liu, Qiongbo Hu, Qunfang Weng, Ke Zhang

**Affiliations:** 1 State Key Laboratory of Green Pesticide, College of Plant Protection, South China Agricultural University, Guangzhou, China; Agroscope, Nyon, Switzerland

**Keywords:** fungal diversity, fungi, herbicidal, biocontrol, soil fungi, biopesticides

## Abstract

Weeds pose a significant threat to agricultural productivity, emphasizing the urgent need for developing innovative biological herbicides. Soil is a rich reservoir of fungi with potential herbicidal properties. The Xinjiang region of China, characterized by unique biodiversity shaped by geographical and climatic factors, is likely to harbor distinctive fungal resources that remain understudied. This study investigated the herbicidal potential of soil fungi by collecting 123 soil samples from 33 diverse habitats in Xinjiang and isolating 114 fungal strains. Morphological characteristics and ITS sequence analysis identified these strains as 24 species belonging to 12 genera. Subsequently, 24 representative strains underwent phytotoxicity assays using detached weed leaves. Strain Tapu14C02 demonstrated significant herbicidal activity against 11 weeds, including *Amaranthus retroflexus*, *Bidens pilosa*, and *Celosia argentea*. Further identification confirmed the strain as *Talaromyces purpureogenus*. Pot experiments were conducted to evaluate the herbicidal potential of the strain. The spore suspension at a concentration of 1.0 × 10^8^ spores/mL inhibited barnyard grass (*Echinochloa crus-galli*) seedling root length by 93.07%. Among the crude extracts from the fermentation broth, the ethyl acetate fraction exhibited the strongest herbicidal activity, causing complete inhibition of root growth at concentrations of 1000 µg/mL and 500 µg/mL. This study provides novel insights into the herbicidal potential of soil fungi in the Xinjiang region of China.

**IMPORTANCE:**

Weeds pose significant challenges by causing agricultural losses and ecological harm. Over the past decades, many weed species have developed high resistance to chemical herbicides, underscoring the urgent need for new biological herbicide alternatives. In this study, we isolated and screened herbicidal fungi from soil samples in Xinjiang with unique conditions of extreme arid. Notably, we discovered the T. purpureogenus strain Tapu14C02, which shows promising potential as a myco-herbicide. Both its conidia and fermentation broth exhibit broad-spectrum effectiveness against weeds. This research highlights the potential of fungal resources for sustainable agriculture.

## INTRODUCTION

Agricultural pests (weeds, insects, mites, plant pathogens, etc.) pose a significant threat to crop production. Without control measures, crop yield losses can exceed 50%, leading to a 50% reduction in wheat production (1) and an 80% loss in cotton (2). Weeds are the most harmful pests, causing potential losses of up to 34% (1, 2). Pesticides play a crucial role in Integrated Pest Management (IPM). According to the Food and Agriculture Organization (FAO), since 2010, the global annual consumption of agricultural pesticides has reached 4 million tons (https://www.fao.org/faostat/en/#data/RP, accessed on 18 May 2024). Since the mid-1970s, the number of weed species resistant to herbicides has increased linearly, creating a high demand for herbicides with new modes of action (MOA). However, since the 1980s, only one herbicide with a new MOA has been introduced to the market (3). The increasing resistance of weeds to herbicides, coupled with the lack of new MOA herbicides, has led some scholars to predict that by 2050, almost all existing herbicides will be ineffective (4).

Many research projects around the world focus on the development and introduction of new environmentally friendly and low-toxicity plant protection products, also known as “biorational plant protection products (BPPP)” (5). In recent years, the trend in global pesticide development has gradually shifted from chemical pesticides to biopesticides due to their lower environmental impact. Fungi play a crucial role in BPPP as they are a significant source of microbial pesticides (6). For instance, *Beauveria bassiana* has long been developed as a well-known fungal insecticide, *Trichoderma harzianum* serves as an important fungicide, and *Purpureocillium lilacinum* is a key nematicide. “Lubao No. 1” (*Colletotrichum acutatum* sp. *cuscuta*), a fungal herbicide, has been used in China for many years. *Phoma macrostoma* is able to control broadleaf weeds in turf and agricultural settings by producing compounds that cause chlorosis and necrosis in susceptible weeds (7, 8). *Sclerotinia minor*, primarily used in the United States to combat dandelions and other broadleaf weeds, is applied in granule form and has proven effective in reducing weed populations without harming target crops (9). However, the application of fungi in BPPP also faces the issue of insufficient resource supply. The strains and compounds currently used were discovered 30 years ago, some even over 200 years ago (10). The lack of new strains and novel structured compounds is a significant factor limiting the development of fungal BPPP.

Unique ecosystems often harbor unique species (11). The Xinjiang region, located in northwestern China, boasts a rich and diverse array of ecosystems, including deserts, glaciers, grasslands, forests, river valleys, and mountains. The distinct geographical location and variable climatic conditions of Xinjiang have created an ecological environment, unlike any other region. These ecosystems provide abundant habitats for soil fungi. More importantly, extreme conditions like saline alkali and dryness make organisms difficult to survive, allowing those species with strong vitality to be preserved. Therefore, this study aims to explore the species composition and distribution of soil fungi in Xinjiang and preliminarily investigate the herbicidal activity of these fungi. The research results will give a new reference to the discovery and utilization of fungal resources and offer new solutions for agricultural pest control.

## MATERIALS AND METHODS

### Collection of soil samples

**Fig 1:**
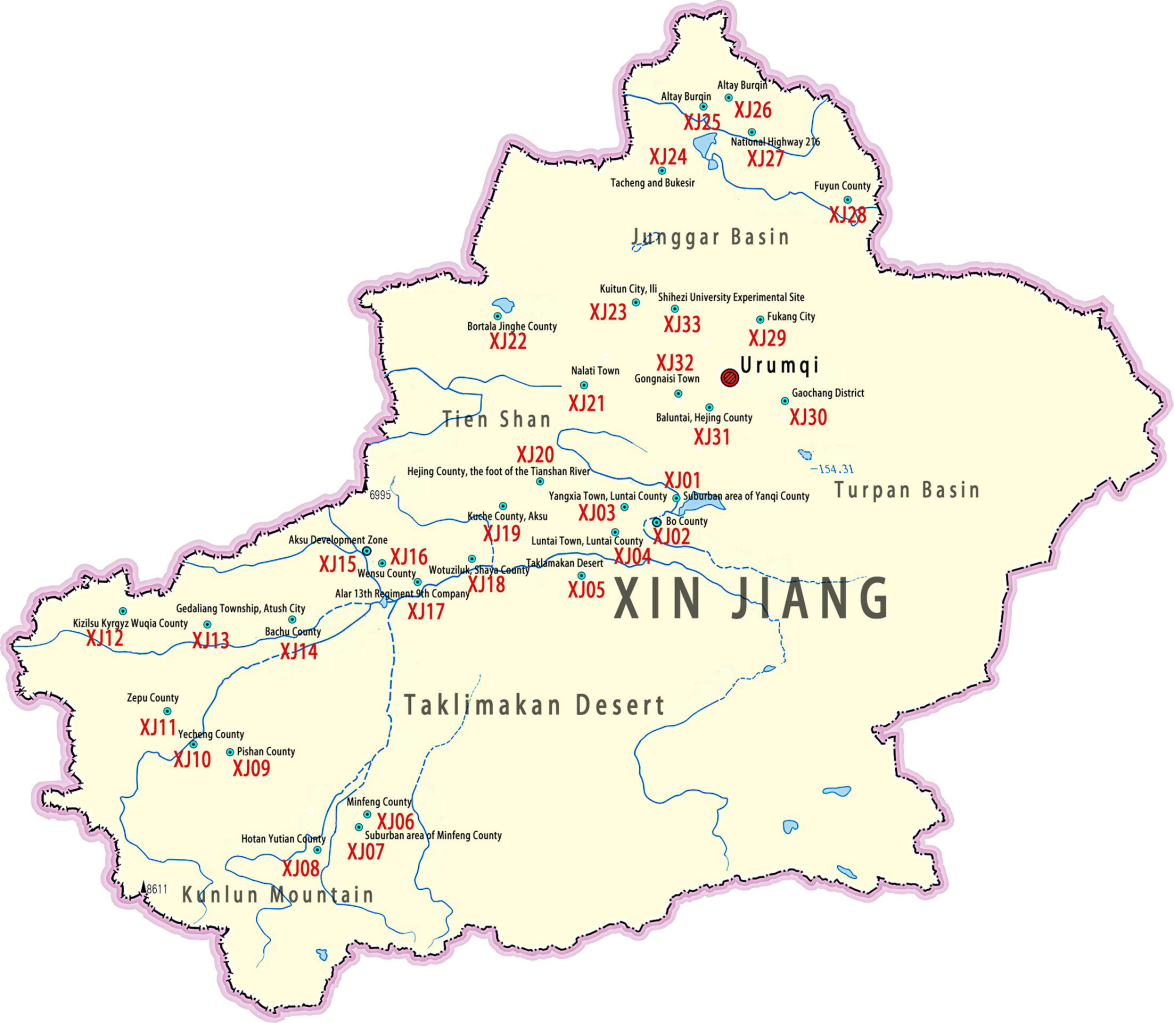
Distribution of sampling locations in Xinjiang. The map was downloaded from the Standard Map Service established by the Map Technology Review Center of the Ministry of Natural Resources, Beijing, China, http://211.159.153.75.

Based on vegetation conditions, we selected sampling sites representing various habitats, including human-cultivated lands such as corn, cotton, vegetable, and orchard, as well as non-cultivated lands such as forest, grass, and desert. The longitude, latitude, and altitude of each sampling site were meticulously recorded using ICEGPS 100C (Shenzhen, China) and marked the sampling points on the Standard Map Service (established by Map Technology Review Center of the Ministry of Natural Resources of China, http://211.159.153.75). During collection, ~100 g of soil was gathered from a depth of ~20 cm at three randomly selected positions within each site. These soil samples were then combined, securely bagged, labeled with corresponding sampling point information, and stored at 4°C. In total, 123 soil samples were collected from 33 distinct locations across the Xinjiang region (Fig. 1; Table S1).

### Isolation and purification of fungal strains

The method described by Chen et al. (12) was referred to with slight modifications. Initially, soil samples were crushed and thoroughly mixed, then passed through a 40-mesh sieve. Subsequently, 10 g of soil was weighed and suspended in 100 mL of 0.10% sterile Tween-80 solution, which was vortexed to ensure thorough mixing. Then, 100 µL suspension was inoculated onto each selective medium plate (potato 200 g, glucose 20 g, agar 20 g, chloramphenicol 50 mg, cycloheximide 50 mg, Bengal rose 50 mg, and distilled water 1 L) and spread evenly. Plates were sealed with parafilm and incubated at 26±1°C. Each soil sample was inoculated onto three plates, and the experiment was repeated three times. Once fungal colonies were observed on the plate, the single colonies were transferred to PDA plates (potato 20 g, glucose 20 g, agar 20 g, and distilled water 1 L) for pure culture. This process was repeated until the strains were purified. In a sterile environment, scrape an appropriate amount of purified isolated fungal hyphae from PDA plates, inoculate them into a 25% glycerol solution, and store them in a laboratory-specific strain storage refrigerator at −80°C.

### Identification of fungal strains

Species identification of fungal strains isolated from soil involved morphological and molecular genetic techniques. Methods described by Chen et al. (12) were followed. Colonies on Potato Dextrose Agar (PDA) plates were characterized by measuring color, diameter, and other features. Microstructures including mycelia, conidia, and sporulation were observed under a BMC 500 light microscope (Phoenix Optical Group Co., Ltd., Jiangxi, China). Genomic DNA was extracted using DP3112 DNA extraction kits (Bio-Teke, Beijing, China), following the manufacturer’s protocol. The Internal Transcribed Spacer (ITS) region of rDNA was amplified using primers ITS1 (5′-TCCGTAGGTGAACCTGCGG-3′) and ITS4 (5′-TCCTCCGCTTATTGATATGC-3′) (13). PCR products were analyzed by 1% agarose gel electrophoresis and sequenced via Sanger sequencing. Sequences obtained were initially identified and phylogenetically analyzed using BLAST comparisons in the NCBI nucleotide database. ITS sequences were aligned and used to construct phylogenetic trees in MEGA 11.0 using the Maximum Likelihood (ML) method with a bootstrap test of 500 replications and the Jukes–Cantor model (14). Standard strain information for phylogenetic analysis is provided in Table S2.

### Phytotoxic assessment of fungal strain disc on detached weed leaf

The leaves were cut from the healthy weed plants and washed with sterile Tween-80 and distilled water to remove the surface microbes. The leaves were placed onto the 1% agar plate (1 g agar powder and 99 g distilled water). Then, picked out a disc from the fungal plate with a 6 mm diameter puncher, and moved the fungal disc onto the detached leaf on a agar plate. The detached leaves were incubated at 70–80% humidity for 7 days. Then, phytotoxicity was assessed by measuring lesion areas and grading them based on the percentage of the lesion area relative to the total leaf area, with grading criteria as follows: grade 1 = 0%; grade 2 = 0-3.0%; grade 3 = 3.0–6.0%; grade 4 = 6.0-12.0%; grade 5 = 12.0–25.0%; and grade 6 > 25.0%. Each treatment was repeated three times. The agar disc was set as control. Select *Erigeron canadensis* as the first test plant for preliminary phytotoxic assessment of isolated strains, and the screened strains will be tested on other weed plants (*Mikania micrantha*, *Portulaca oleracea*, *Setaria viridis,* etc) by the same method.

### Study on *Talaromyces purpureogenus* strain Tapu14C02

#### Further identification

Further identification involved both morphological characterization and DNA barcode analysis. The fungal strains were cultured on PDA and MEA plates (malt extract powder 130 g, agar 15 g, chloramphenicol 0.1 g, and distilled water 1L). Sporulation structures were examined using scanning electron microscopy (Regulus 8100 SEM, Hitachi High-Tech Co., Ltd., Shanghai, China). DNA extracts were subjected to PCR amplification using specific primers: Bt2a (5′-GGTAACCAAATCGGTGCTGCTTTC-3′) and Bt2b (5′-ACCCTCAGTGTAGTGACCCTTGGC-3′) (15) for the beta-tubulin (BenA) gene; the primers Cmd5 (5′-CCGAGTACAAGGARGCCTTC-3′) and Cmd6 (5′-CCGATRGAGGTCATRACGTGG-3′) (15) for the calmodulin (CaM) gene. Phylogenetic trees were constructed based on the DNA sequences of ITS, BenA, and CaM, following the methods detailed in Section 2.3 for DNA barcode analysis.

#### Effects of conidia on seedling growth of *E. crus-galli*

The herbicidal activity of Tapu14C02 conidia was evaluated using the little cup test. Conidia were harvested from a PDA plate colony and suspended in a 0.10% sterile Tween-80 solution to achieve a final concentration of 1.0 × 10^8^ spores/mL. Add 5 mL spore suspension to plastic cups lined with glass beads and gauze for moisture retention. Each cup contained 10 well-germinated *E. crus-galli* (barnyard grass) seeds, and the cups were then incubated in a climate chamber at 26±1°C and 50% relative humidity for 7 days. The control group was treated with 0.10% sterile Tween-80 solution, with five replicates per treatment. After 7 days, the lengths of both the stems and roots of *E. crus-galli* seedlings were measured. The inhibition rate of seedling growth by the spore suspension was calculated using the formula:

Inhibition rate (%) =100% × (control seedling length−treated seedling length)/control seedling length.

#### Effects of fermentation broth on weeds in pot-test

Tapu14C02 was cultured on PDA plates for 7–14 days. Conidia were then collected and inoculated into 1000 mL of PD liquid medium (200 g/L potato, 20 g/L glucose) at a concentration of approximately 1.0 × 10^6^ spores/mL for 2–3 days. Subsequently, the seed broth was transferred to 7000 mL of PD broth for further culture amplification at 28 ± 1°C and 160 rpm for 7 days. The fermentation broth was obtained by filtering through gauze to remove residues.

For the bioassay, post-tests were conducted using a foliar spray method following the guidelines outlined in "Indoor Bioassay Guidelines for Herbicides" (NY/T 1155.4–2006) issued by the Ministry of Agriculture and Rural Affairs of China. In brief, the clean square pots (8 cm × 8 cm) were prepared and filled with clean soil containing less than 3% organic matter, pH7, good permeability, and uniform texture to 4/5 of the pot volume. Water trays were placed beneath each pot to ensure soil moisture through capillary action.

Pre-germinated seeds of *Echinochloa crus-galli*, *Amaranthus retroflexus*, *Digitaria sanguinalis*, and *Celosia argentea* were evenly sown into pots containing clean soil with a thickness of 0.5–1 cm and incubated in a light incubator at 26 ± 1°C until reaching the 4–6 leaf stage suitable for bioassays. Each pot of weeds received an even application of 2 mL of fermentation broth using a quantitative sprayer, with three replicates per weed type. PD liquid medium served as the control. The pots were then incubated in a light incubator at 26 ± 1°C for 7 days, during which the plants were monitored for color changes (chlorosis, yellowing, and bleaching), morphological changes (leaf curling, deformities, and lesions), and growth changes (wilting, withering, and clustering). These observations were compared with those of the control groups.

#### Effects of crude extracts of fermentation broth on seedling growth of *E. crus-galli*

The filtered fermentation broth was mixed with equal volumes of organic solvents including n-butanol, ethyl acetate, dichloromethane, and petroleum ether. The mixture underwent ultrasonic extraction at 40 kHz for 40 minutes, repeated three times. The organic phase was collected and concentrated by rotary evaporation to obtain crude extracts. These extracts were then dissolved in DMSO to prepare stock solutions at 5000 µg/ml concentration, which were subsequently diluted with Tween-80 solution to prepare working solutions for the bioassay. The bioassay method followed the procedures outlined in Section 2.5.2.

### Data analysis

The experimental data were analyzed and visualized using Excel 2010 (Microsoft, USA). Meanwhile, Duncan’s Multiple Range Test (DMRT ) was used to compare the difference of means by employing SPSS 25 (IBM Company, USA).

## RESULTS

### Isolation and identification of soil sample strains

**Fig 2:**
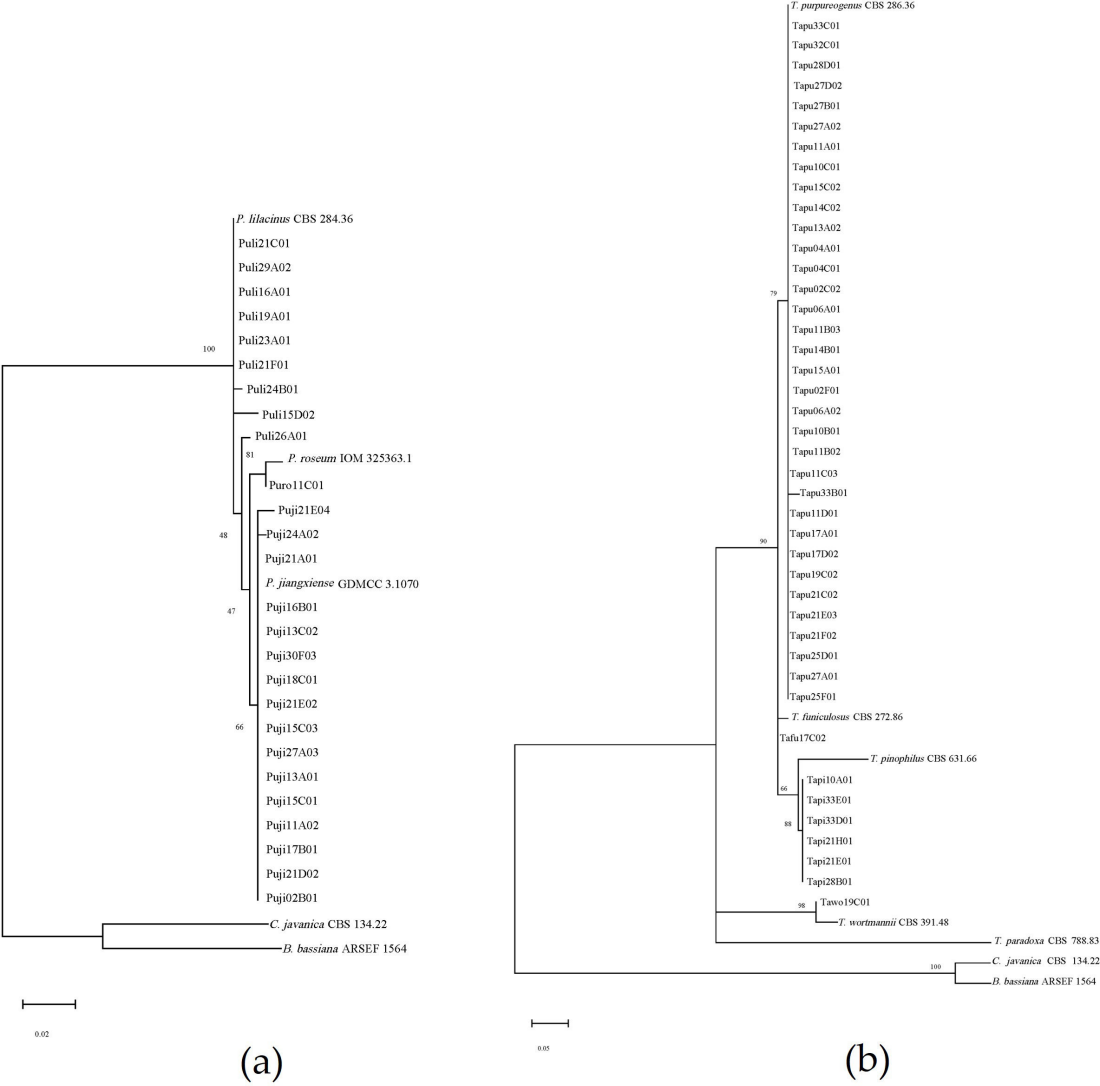
Phylogenetic trees of *Purpureocillium* and *Talaromyces* genera based on ITS sequence. (**a**) *Purpureocillium* genus and (**b**) *Talaromyces* genus.

**Fig 3:**
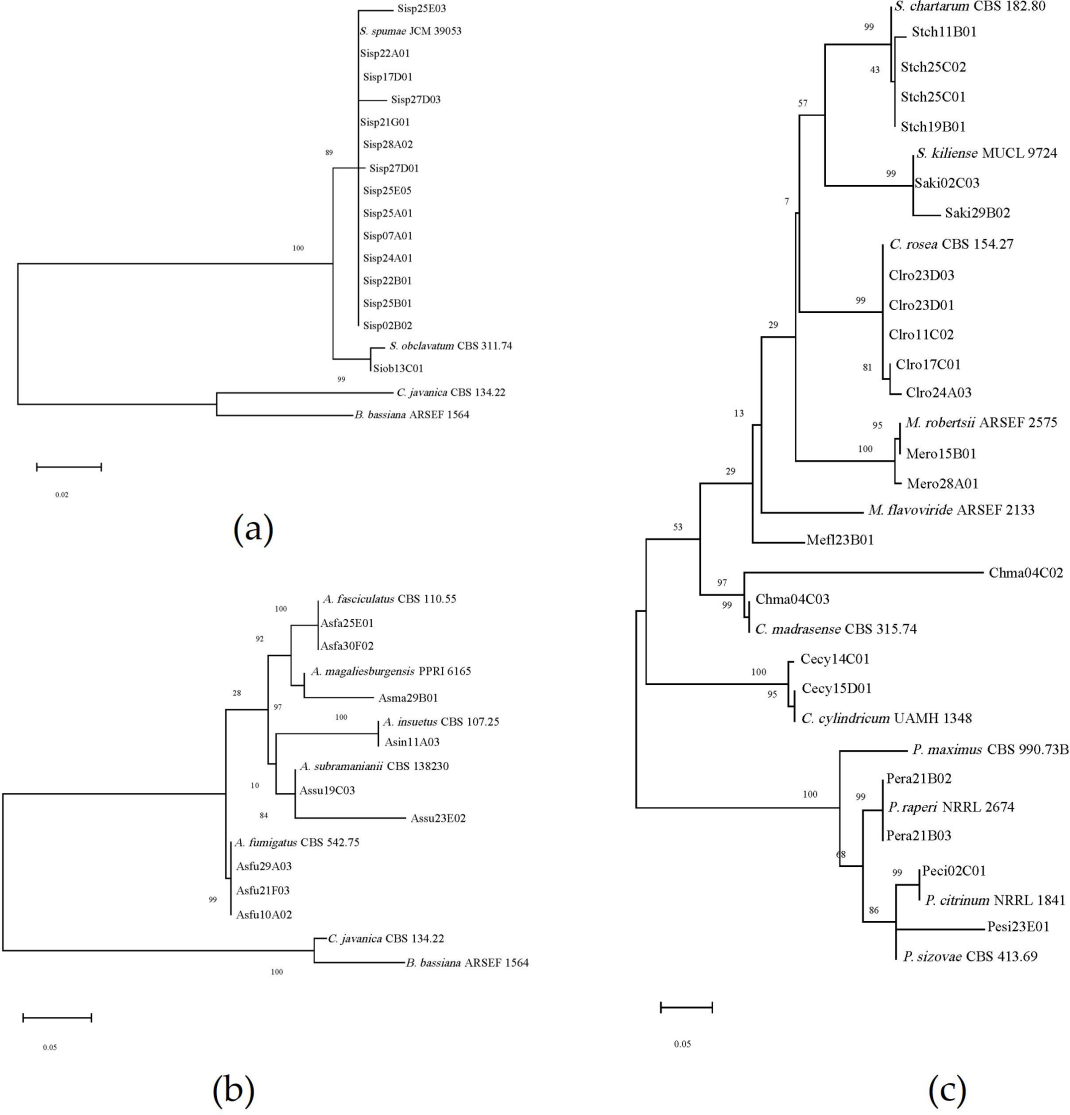
Phylogenetic trees of *Aspergillus*, *Simplicillium*, and other genera based on ITS sequence. (**a**) *Simplicillium* genus, (**b**) *Aspergillus* genus, and (**c**) others.

A total of 123 soil samples were collected from 33 locations across the Xinjiang region (Table S1). From these samples, 114 fungal strains were isolated, resulting in an isolation rate of 56.10%, with an average of 0.93 strains per soil sample. Preliminary morphological identification (Fig S1) and phylogenetic analysis based on ITS sequences (Fig. 2 and 3) revealed that these strains belong to 12 genera and 24 species. Among them, the genus *Aspergillus* comprised five species, while *Purpureocillium* and *Talaromyces* each comprised four species. Other genera had 1–3 species.

Notably, *T. purpureogenus* was the most abundant species, with 34 strains accounting for 29.82% of the total isolates, followed by *Purpureocillium jiangxiense* with 16 strains, representing 14.04%.

The isolation of fungi varied with different habitats, as summarized in Table 1. Excluding desert soil samples, other habitats yielded between 52% and 85% isolation rates, resulting in 0.7–1.4 fungal strains and 0.3–0.6 species per sample. The isolation rates, ranked from highest to lowest, were as follows: Vegetable >Grass > Corn >Orchard > Forest >Cotton > Desert. Vegetable soil samples exhibited the highest isolation rate, whereas desert soil samples showed the lowest, with no fungal strains isolated. Furthermore, orchard soil samples yielded the highest number of fungal strains and species, with 27 strains and 12 species identified, the forest followed with 24 strains and 11 species, and the grass with 23 strains and 10 species.

**TABLE 1:** Fungal isolation of Xinjiang soil samples^*a*^

Habitat	Soil sample			Fungi strain		Fungi species	
	Numbers	Fungi (+)	Fungi (+) (%)	Number	Mean/sample	Number	Mean/sample
Corn	8	5	62.500	6	0.750	3	0.375
Cotton	19	10	52.632	17	0.895	8	0.421
Orchard	21	13	61.905	27	0.129	12	0.571
Vegetable	13	11	84.615	17	1.308	7	0.538
Forest	29	17	58.621	24	0.828	11	0.379
Grass	17	13	76.471	23	1.353	10	0.588
Desert	16	0	0.000	0	0.000	0	0.000
Total	123	69	56.098	114	0.927	24	

^*a*^ Fungi (+), the soil samples isolated fungi.

### Phytotoxic activity of fungal hyphae disc to detached weed leaves

The phytotoxic effects of 24 fungal strains selected from different species on *E. canadensis* leaves were assessed (Table 2). After a 7-day inoculation period, significant phytotoxic activity was observed in the *T. purpureogenus* strain Tapu14C02, resulting in a lesion grade of 6. In contrast, the remaining strains showed minimal effects on *E. canadensis* leaves. Consequently, Tapu14C02 was chosen for further research.

**TABLE 2:** Phytotoxic activity of tested strains on the detached leaves of *E. canadensis^a^*

Fungal strain ID	Fungal species	Lesion grade
Tapu14C02	*Talaromyces purpureogenus*	6
Tapi10A01	*Talaromyces pinophilus*	1
Tafu17C02	*Talaromyces funiculosus*	1
Tawo19C01	*Talaromyces wortmannii*	3
Puji21E04	*Purpureocillium jiangxiense*	1
Puli21C01	*Purpureocillium lilacinus*	2
Puro11C01	*Purpureocillium roseum*	1
Asfu21F01	*Aspergillus fumigatus*	3
Asfa25E01	*Aspergillus fasciculatus*	1
Assu23E02	*Aspergillus subramanianii*	1
Asin11A03	*Aspergillus insuetus*	1
Asma29B01	*Aspergillus magaliesburgensis*	1
Sisp17D01	*Simplicillium spumae*	1
Siob13C01	*Simplicillium obclavatum*	1
Pesi23E01	*Penicillium sizovae*	1
Peci02C01	*Penicillium citrinum*	1
Pera21B02	*Penicillium raperi*	1
Mefl23B01	*Metarhizium flavoviride*	1
Mero15B01	*Metarhizium robertsii*	1
Clro23D01	*Clonostachys rosea*	1
Stch25C01	*Stachybotrys chartarum*	1
Saki02C03	*Sarocladium kiliense*	2
Chma04C02	*Chaetomium madrasense*	1
Cecy15D01	*Cephalotrichum cylindricum*	1
CK		1

^*a*^ CK, control.

**Fig 4:**
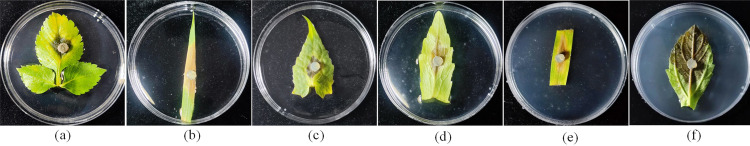
Phytotoxic activity of Tapu14C02 strain on detached weed leaves. (**a**) *B. pilosa*, (**b**) *D. sanguinalis*, (**c**) *M. micrantha*, (**d**) *E. canadensis*, (**e**) *S. viridis*, and (**f**) *S. calendulacea*.

Notably, the Tapu14C02 strain displayed broad-spectrum phytotoxicity in subsequent experiments, damaging detached leaves from 12 weed species as shown in Table 3. The lesions on some of the weed leaves spread from the infection site to adjacent areas (Fig. 4).

**TABLE 3:** Phytotoxic effects of Tapu14C02 strain on the detached leaves of weed species

Weed species	Lesion grade at 7 days
*Amaranthus retroflexus*	6
*Alternanthera philoxeroides*	6
*Bidens pilosa*	6
*Celosia argentea*	6
*Digitaria sanguinalis*	6
*Erigeron canadensis*	6
*Echinochloa crus-galli*	6
*Mikania micrantha*	6
*Portulaca oleracea*	6
*Setaria viridis*	6
*Spermacoce pusilla*	6
*Sphagneticola calendulacea*	6

### Further identification of Tapu14C02 Strain

**Fig 5:**
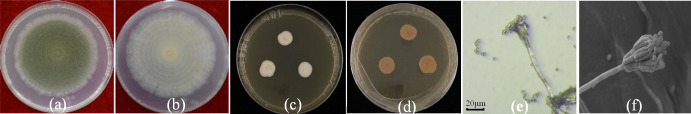
Morphological characteristics of Tapu14C02 strain. (**a**)/(**b**) The front/back view of a single colony of Tapu14C02 strain on PDA plate after 7 days; (**c**)/(**d**) the front/back view of a single colony of Tapu14C02 strain on MEA plate after 7 days; (**e**) a microscopic profile of conidia and sporulation structure; and (**f**) sporulation structure of the strain captured by SEM.

**Fig 6:**
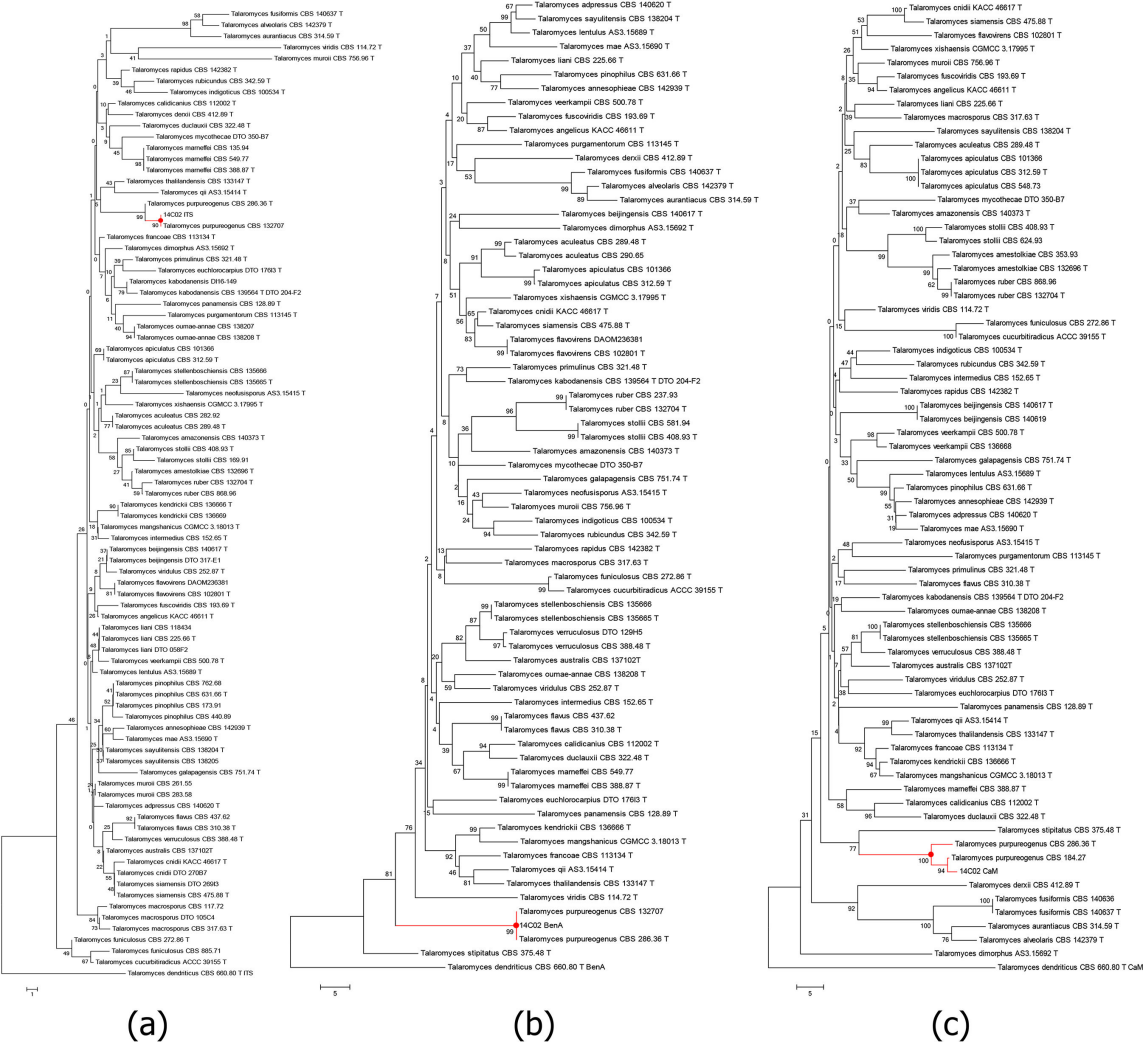
Phylogenetic trees of Tapu14C02 strain based on the sequences of ITS (**a**), BenA (**b**), and CaM (**c**).

The strain Tapu14C02 was further identified as *T. purpureogenus* through morphological and molecular genetic analyses (Fig. 3 and 4). The strain grew quickly on a PDA plate at 25℃, and the colony diameter was 7.7 ± 0.2 cm after 7 days of treatment, with white mycelia and the green conidia layer (Fig. 5a and b). In comparison, the strain plates had a different colony texture and slow growth on MEA. The colony diameter was 1.2 ± 0.3 cm after 7 days of treatment. Initially, colonies were white with fewer conidia, later transitioning to a blue-green appearance with a yellow-brown reverse, and producing numerous spores with abundant aerial hyphae (Fig. 3c and d). Morphologically, the strain’s conidiophores were strictly biverticillate without subterminal branches; stipes were smooth-walled; metulae were arranged in verticils of 3–5; phialides acerose, typically 3–6 per metula; and conidia were smooth, ellipsoidal, measuring 3–3.5 × 2–2.5 µm (Fig. 5e and f). In summary, these morphological features were consistent with the description of type strain (16). The phylogenetic trees were constructed based on the sequences of ITS, BenA, and CaM (Fig. 6; Table S3), which supported absolutely that the strain belongs to the species, *T. purpureogenus*.

### Herbicidal effect of Tapu14C02 conidia on the seedlings of *E. crus-galli*

**Fig 7:**
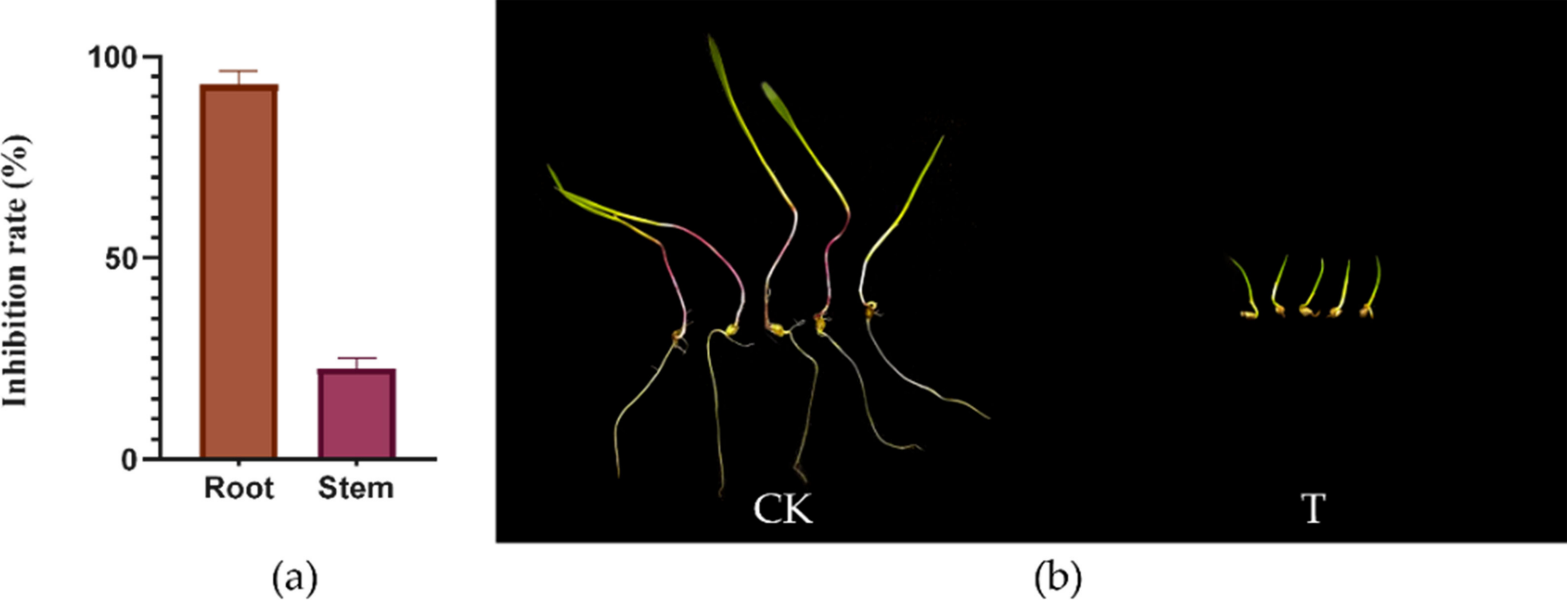
Effects of Tapu14C02 conidia on the seedlings of *E. crus-galli*. (**a**) The inhibition rate on root and stem lengths of *E. crus-galli* seedlings 7 days after applying the Tapu14C02 spore suspension. (**b**) Profiles of *E. crus-galli* seedlings. CK: control and T: treatment.

The inhibitory effects of Tapu14C02 on the growth of *E. crus-galli* seedlings were clearly observed (Fig. 7). Following treatment with a concentration of 1.0 × 10^8^ spores/mL for 7 days, the relative inhibition rates of root and stem lengths were significant. Specifically, the root length was inhibited by 93.07%, indicating a profound impact on root development. In contrast, the inhibition of stem length was less pronounced, with a relative inhibition rate of 22.65%. These results underscore the potent herbicidal activity of Tapu14C02 against *E. crus-galli*, particularly affecting root growth.

### Herbicidal activity of fermentation broth of Tapu14C02 strain in pot-test

**Fig 8:**
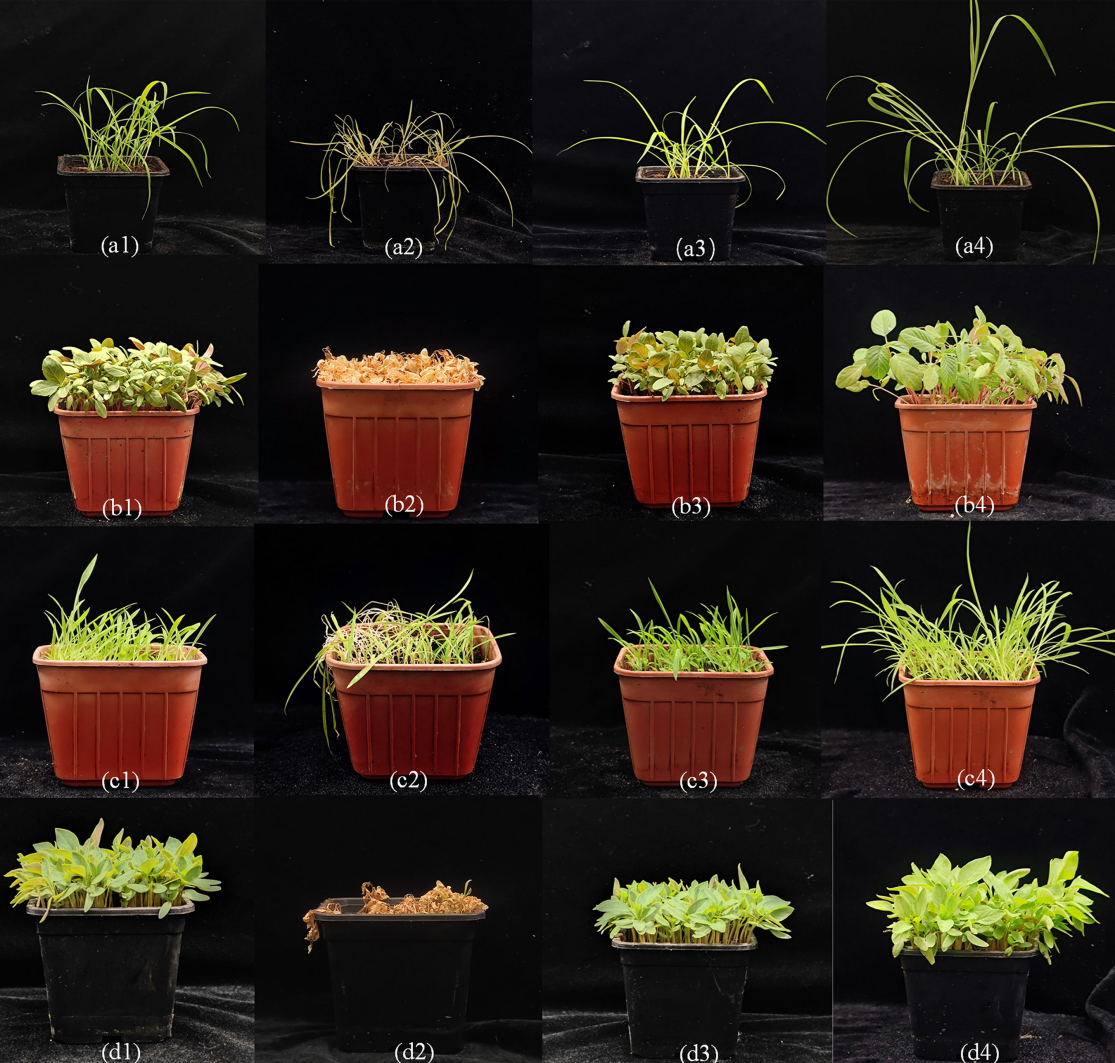
Effects of fermentation broth of Tapu14C02 on the weed growth in pot-test. (a) *E. crus-galli*; (b) *A. retroflexus*; (c) *D. sanguinalis*; and (d) *C. argentea*. (1) and (2) at the 0 and 7 days after treatment by Tapu14C02; (3) and (4) at the 0 and 7 days in control.

The fermentation broth of the Tapu14C02 strain demonstrated robust herbicidal activity against four different tested weed species (Fig. 8). Following 7 days of treatment, the weeds treated with Tapu14C02 fermentation broth displayed notable symptoms including poor growth, wilting, and in severe cases, complete plant death. The leaves of treated plants exhibited chlorosis (yellowing) and lesions, contrasting starkly with the healthy appearance of the control group plants (Fig. 8). These observations highlight the potent herbicidal effects of Tapu14C02 fermentation broth on monocotyledonous and dicotyledonous weeds.

### Effects of crude extracts from Tapu14C02 fermentation broth on the growth of *E. crus-galli*

**Fig 9:**
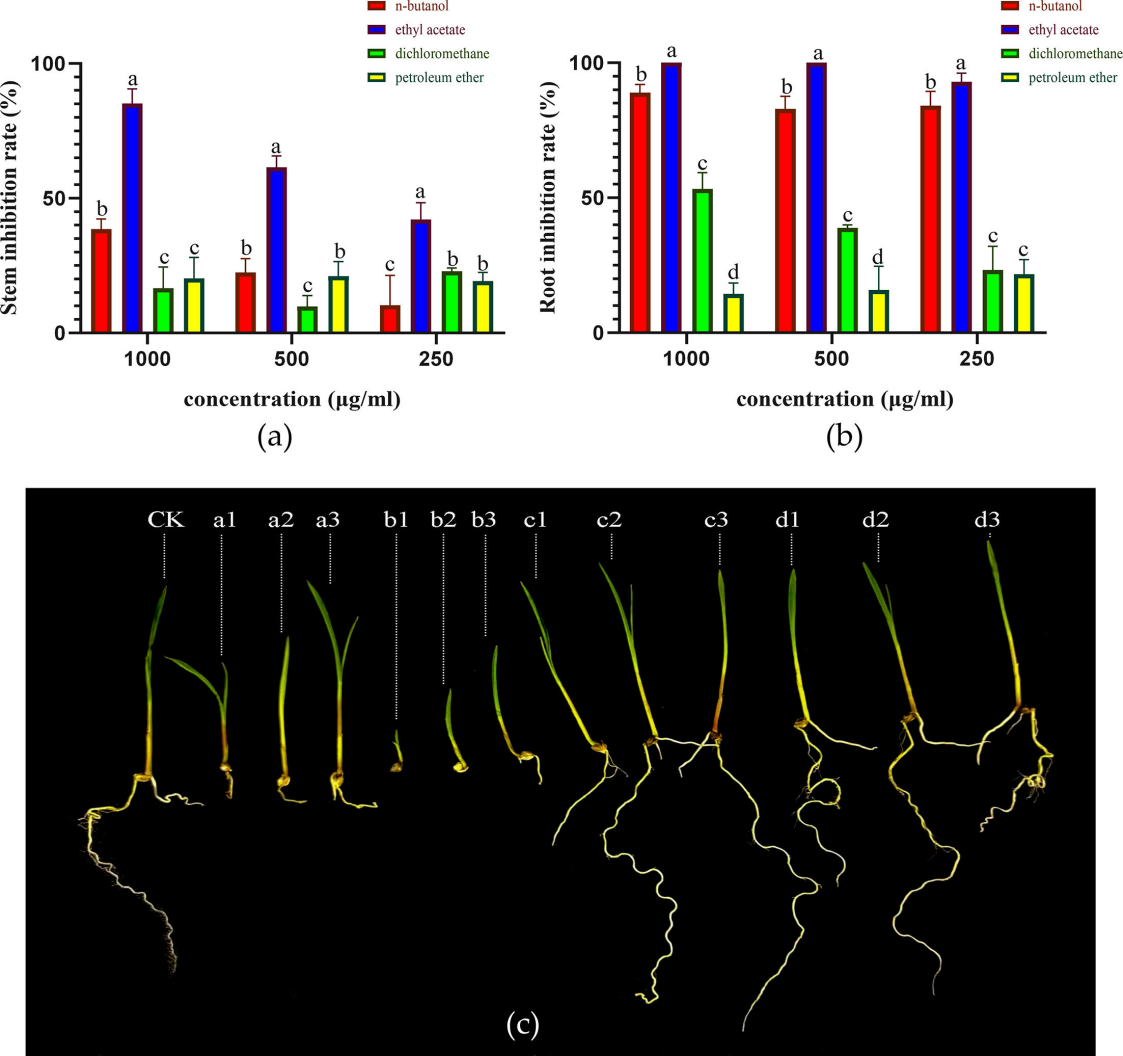
Inhibitory effects of different crude extracts and concentrations on the growth of *E. crus-galli* roots and stems. (**a**) Inhibition rates of crude extracts on the stem growth of *E. crus-galli*; (**b**) inhibition rates of crude extracts on the root growth of *E. crus-galli*. The different letters on the bar chart at the same concentration indicate significant differences as determined by DMRT (*P* > 0.05). The inhibition rate shown in the figure is the average of five repeated treatments; and (**c**) seedling profiles *E. crus-galli* in each treatment group. **a1/2/3,** n-butanol extracts at 1000/500/250 µg/mL, respectively; **b1/2/3,** ethyl acetate extracts at 1000/500/250 µg/mL, respectively; **c1/2/3,** dichloromethane extracts at 1000/500/250 µg/mL; and **d1/2/3,** petroleum ether extracts at 1000/500/250 µg/mL.

The inhibitory effects of extracts from the fermentation broth of the Tapu14C02 strain on the growth of *E. crus-galli* seedlings were evaluated using four different organic solvents (Fig. 9). The results indicated significant variation in herbicidal activity among the solvents tested. The herbicidal activity ranked from highest to lowest as follows: ethyl acetate >n-butanol >dichloromethane > petroleum ether (Fig. 9a and b). The crude extract of the ethyl acetate exhibited the strongest herbicidal activity with 100% inhibition of root growth and over 60% inhibition of stem growth at the concentrations of 1000 µg/mL and 500 µg/mL. The n-butanol extract also showed effective herbicidal activity, although slightly less potent than that of ethyl acetate, and the inhibition rate on root growth was more pronounced than on stem length (Fig. 9c). The crude extracts from dichloromethane and petroleum ether did not exhibit significant inhibitory effects compared to ethyl acetate and n-butanol (Fig. 9c). These findings highlight the superior herbicidal potential of the ethyl acetate extract from Tapu14C02 fermentation broth.

## DISCUSSION

In this study, 114 strains belonging to 24 species were isolated from 123 soil samples, indicating a relatively low isolation rate. This could be attributed to two main factors: the limited biodiversity in Xinjiang and the methodologies employed in our research. The extreme natural conditions in Xinjiang, including dryness, salinization, alkalization, and large temperature variations, constrain the development of organisms, thereby reducing biodiversity. Additionally, the use of selective media for isolation may have restricted the discovery of fungal strains. Furthermore, reliance solely on the ITS sequence for species identification likely influenced species recognition. Nonetheless, this study offers valuable insights into the diversity of soil fungi in the Xinjiang region.

Unlike the southern, central, and southwestern regions of China, where *P. lilacinum* is the most abundant species (12, 17, 18), the largest numbers of fungal strains isolated from soil samples in the Xinjiang region were *T. purpureogenus* and *P. jiangxiense*, which suggests their adaptability to Xinjiang’s unique climate and environment. *P. jiangxiense* was discovered in Jingxi Province of China by our team, which is an entomopathogenic fungus (19). Despite Xinjiang having the most extensive sampling area and the highest number of soil samples collected compared to Southwest and Southern China (12, 17), it exhibited the lowest fungal isolation rate.

Interestingly, strains of *T. purpureogenus* primarily originate from artificially managed vegetation types closely associated with local agriculture. Previous studies have demonstrated that certain isolates of *T. purpureogenus* can effectively suppress the growth of diverse crop pathogens and modulate the composition of soil microbial communities (20). Investigating the interactions between these strains and crop pathogens is crucial for understanding why *T. purpureogenus* has become prevalent in agricultural regions of Xinjiang.

In addition to its recognized antibacterial and insecticidal properties (21), we are the first to report its herbicidal activity. This finding holds significant implications for integrating *T. purpureogenus* into field management strategies, addressing crop pathogens that utilize field weeds and pests as intermediate hosts or transmission vectors. Despite limited research on the interaction between *T. purpureogenus* and plants, relevant studies indicate that it acts as a drought-tolerant endophytic fungus capable of promoting crop growth. This enhances various physiological and biochemical traits in wheat seedlings under both normal and stress conditions (22). These findings also provide pertinent references for further investigating the safety of herbicidal products developed from *T. purpureogenus* and its secondary metabolites for crops.

In recent years, *E. crus-galli* has developed resistance to chemical herbicides in rice fields (23, 24). Choosing *E. crus-galli* as an experimental subject to investigate further the herbicidal activity of *T. purpureogenus* holds both theoretical significance and practical application value.

In studying the herbicidal activity of the *T. purpureogenus* strain, we employed both detached leaf and whole-plant bioassays to verify its efficacy. This method has been proven more reliable than single detached leaf screening by several studies. The results showed that *T. purpureogenus* has high activity against various agricultural and invasive weeds and is effective against both monocotyledonous and dicotyledonous weeds, highlighting its broad applicability. We tested the herbicidal activity of *T. purpureogenus* using both spore suspensions and crude fermentation extracts, applying them as bioherbicides and secondary metabolite-based herbicides, respectively. The results showed that the conidial suspension of *T. purpureogenus* at a concentration of 1.0 × 10^8^ spores/mL achieved an inhibition rate of 93%. Notably, the fermentation extract of *T. purpureogenus* exhibited superior herbicidal activity, inducing significant mortality in living weed plants without causing mechanical damage. Moreover, it achieved an inhibition rate of over 90% on the root growth of barnyard grass seedlings at relatively low concentrations of its ethyl acetate extract. It was reported that the phytotoxin dirhamnolipid (Rha-Rha-C10-C10) from the ethyl acetate extract of fermentation broth of the herbicidal fungus *Colletotrichum gloeosporioides* achieved an inhibition rate of approximately 91% on barnyard grassroots at a concentration of 1000 µg /L (25), which is lower than the inhibition rate achieved by the ethyl acetate extract of *T. purpureogenus* fermentation broth at a concentration of 250 µg/L in our study. These findings collectively suggest that the strain Tapu14C02 exhibits promising potential as a myco-herbicide, warranting further in-depth research for its practical application.

### Conclusions

In conclusion, this study isolated numerous fungal strains from Xinjiang, contributing to our understanding of fungal diversity in soil under adverse environmental conditions. Particularly, we discovered the herbicidal activity of the *T. purpureogenus* strain Tapu14C02. This strain exhibits broad-spectrum phytotoxicity against weeds, demonstrating substantial herbicidal activity in both its hyphae and conidia, as well as in the extracts of its fermentation broth. These findings highlight its potential as a myco-herbicide. These findings deepen our understanding of the role of fungi in ecosystems and provide a theoretical basis for harnessing microbial resources to promote sustainable agriculture.

## Data Availability

All relevant data are within the manuscript.

## SUPPLEMENTAL MATERIAL

Supplemental material spectrum.01589-24-s0001.docx Tables S1 to S3; Fig. S1.

ASM does not own the copyrights to Supplemental Material that may be linked to, or accessed through, an article. The authors have granted ASM a non-exclusive, world-wide license to publish the Supplemental Material files. Please contact the corresponding author directly for reuse.
