# Supplementary material for: Herbicidal fungal strain isolated from soil in Xinjiang, China
Source: Microbiol Spectr. 2024 Oct 17;12(12):e01589-24. doi: 10.1128/spectrum.01589-24 (PMC11619413; doi:10.1128/spectrum.01589-24)
Supplement: Supplemental material — Tables S1 to S3; Fig. S1. [file spectrum.01589-24-s0001.docx]

**Supplementary Materials**

Table S1 Information on soil sampling sites and isolated fungal strains in Xinjiang

| **Longitude & latitude** | **Altitude (m)** | **Administrative area** | **Sample NO.** | **Habitat** | **Strain NO** |
| --- | --- | --- | --- | --- | --- |
| E86.53,N42.02 | 1057.4 | Yanqi County, Bayingolin | 01A | Vegetable |  |
|  |  |  | 01B | Forest |  |
|  |  |  | 01C | Forest |  |
| E86.33,N41.80 | 1057 | Bo County, Bayingolin | 02A | Forest |  |
|  |  |  | 02B | Orchard | Puji02B01 |
|  |  |  |  |  | Sisp02B02 |
|  |  |  | 02C | Cotton | Peci02C01 |
|  |  |  |  |  | Tapu02C02 |
|  |  |  |  |  | Saki02C03 |
|  |  |  | 02D | Corn |  |
|  |  |  | 02E | Desert |  |
|  |  |  | 02F | Forest | Tapu02F01 |
| E84.53,N41.92 | 992.1 | Luntai County, Bayingolin | 03A | Orchard |  |
|  |  |  | 03B | Cotton |  |
|  |  |  | 03C | Desert |  |
|  |  |  | 03D | Desert |  |
| E84.30,N41.72 | 967.7 | Luntai County, Bayingolin | 04A | Vegetable | Tapu04A01 |
|  |  |  | 04B | Desert |  |
|  |  |  | 04C | Cotton | Tapu04C01 |
|  |  |  |  |  | Chma04C02 |
|  |  |  |  |  | Chma04C03 |
|  |  |  | 04D | Forest |  |
|  |  |  | 04E | Forest |  |
|  |  |  | 04F | Forest |  |
| E84.30,N40.75 | 927.3 | Taklamakan Desert in Xinjiang | 05A | Desert |  |
| E82.81,N37.34 | 1348.7 | Minfeng County, Hotan | 06A | Grass | Tapu06A01 |
|  |  |  |  |  | Tapu06A02 |
|  |  |  | 06B | Cotton |  |
| E82.69,N37.02 | 1464.6 | Minfeng County, Hotan | 07A | Forest | Sisp07A01 |
| E81.85,N36.80 | 1461.1 | Yutian County, Hotan | 08A | Orchard |  |
|  |  |  | 08B | Desert |  |
| E78.33,N37.52 | 1407.7 | Pishan County, Hotan | 09A | Forest |  |
|  |  |  | 09B | Desert |  |
|  |  |  | 09C | Cotton |  |
| E77.53,N37.86 | 1345.6 | Yecheng County, Kashgar | 10A | Orchard | Tapi10A01 |
|  |  |  |  |  | Asfu10A02 |
|  |  |  | 10B | Cotton | Tapu10B01 |
|  |  |  | 10C | Orchard | Tapu10C01 |
| E77.27,N38.22 | 1262.7 | Zepu County, Kashgar | 11A | Orchard | Tapu11A01 |
|  |  |  |  |  | Puji11A02 |
|  |  |  |  |  | Asin11A03 |
|  |  |  | 11B | Grass | Stch11B01 |
|  |  |  |  |  | Tapu11B02 |
|  |  |  |  |  | Tapu11B03 |
|  |  |  | 11C | Forest | Puro11C01 |
|  |  |  |  |  | Clro11C02 |
|  |  |  |  |  | Tapu11C03 |
|  |  |  | 11D | Corn | Tapu11D01 |
|  |  |  | 11E | Orchard |  |
| E75.02,N39.74 | 2356.7 | Wuqia County, Kizilsu Kyrgyz | 12A | Desert |  |
|  |  |  | 12B | Forest |  |
| E76.63,N39.77 | 1211 | Atush City, Kizilsu Kyrgyz | 13A | Cotton | Puji13A01 |
|  |  |  |  |  | Tapu13A02 |
|  |  |  | 13B | Desert |  |
|  |  |  | 13C | Forest | Siob13C01 |
|  |  |  |  |  | Puji13C02 |
| E78.49,N39.80 | 1131 | Bachu County, Kashgar | 14A | Forest |  |
|  |  |  | 14B | Grass | Tapu14B01 |
|  |  |  | 14C | Cotton | Cecy14C01 |
|  |  |  |  |  | Tapu14C02 |
|  |  |  | 14D | Desert |  |
| E80.12,N41.21 | 1135.6 | Aksu Development Zone | 15A | Vegetable | Tapu15A01 |
|  |  |  | 15B | Vegetable | Mero15B01 |
|  |  |  | 15C | Grass | Puji15C01 |
|  |  |  |  |  | Tapu15C02 |
|  |  |  |  |  | Puji15C03 |
|  |  |  | 15D | Forest | Cecy15D01 |
|  |  |  |  |  | Puli15D02 |
| E80.40,N41.04 | 1089.9 | Wensu County, Aksu | 16A | Forest | Puli16A01 |
|  |  |  | 16B | Cotton | Puji16B01 |
| E81.55,N40.54 | 996 | 13^th^ Regiment, Alar | 17A | Forest | Tapu17A01 |
|  |  |  | 17B | Orchard | Puji17B01 |
|  |  |  | 17C | Grass | Clro17C01 |
|  |  |  |  |  | Tafu17C02 |
|  |  |  | 17D | Cotton | Sisp17D01 |
|  |  |  |  |  | Tapu17D02 |
| E82.55,N41.27 | 979 | Shaya County, Aksu | 18A | Orchard |  |
|  |  |  | 18B | Cotton |  |
|  |  |  | 18C | Corn | Puji18C01 |
| E83.05,N42.1 | 1498 | Kuche County, Aksu | 19A | Grass | Puli19A01 |
|  |  |  | 19B | Forest | Stch19B01 |
|  |  |  | 19C | Orchard | Tawo19C01 |
|  |  |  |  |  | Tapu19C02 |
|  |  |  |  |  | Assu19C03 |
| E83.59,N42.57 | 2806 | Hejing County, Bayingolin | 20A | Grass |  |
| E83.96,N43.34 | 1332.9 | Xinyuan County, Ili | 21A | Grass | Puji21A01 |
|  |  |  | 21B | Corn | Pera21B02 |
|  |  |  |  |  | Pera21B03 |
|  |  |  | 21C | Forest | Puli21C01 |
|  |  |  |  |  | Tapu21C02 |
|  |  |  | 21D | Corn | Puji21D02 |
|  |  |  | 21E | Orchard | Tapi21E01 |
|  |  |  |  |  | Puji21E02 |
|  |  |  |  |  | Tapu21E03 |
|  |  |  |  |  | Puji21E04 |
|  |  |  | 21F | Orchard | Puli21F01 |
|  |  |  |  |  | Tapu21F02 |
|  |  |  |  |  | Asfu21F03 |
|  |  |  | 21G | Forest | Sisp21G01 |
|  |  |  | 21H | Orchard | Tapi21H01 |
| E82.08,N44.63 | 410.1 | Jinghe County, Bortala | 22A | Cotton | Sisp22A01 |
|  |  |  | 22B | Forest | Sisp22B01 |
| E84.88,N44.57 | 366.7 | Kuitun City, Ili | 23A | Forest | Puli23A01 |
|  |  |  | 23B | Orchard | Mefl23B01 |
|  |  |  | 23C | Orchard |  |
|  |  |  | 23D | Grass | Clro23D01 |
|  |  |  |  |  | Clro23D03 |
|  |  |  | 23E | Grass | Peci23E01 |
|  |  |  |  |  | Assu23E02 |
| E86.52,N46.97 | 1255.8 | Bukesir County, Tacheng | 24A | Grass | Sisp24A01 |
|  |  |  |  |  | Puji24A02 |
|  |  |  |  |  | Clro24A03 |
|  |  |  | 24B | Grass | Puli24B01 |
| E86.82,N47.71 | 463.7 | Burqin County, Altay | 25A | Grass | Sisp25A01 |
|  |  |  | 25B | Forest | Sisp25B01 |
|  |  |  | 25C | Vegetable | Stch25C01 |
|  |  |  |  |  | Stch25C02 |
|  |  |  | 25D | Corn | Tapu25D01 |
|  |  |  | 25E | Vegetable | Asfa25E01 |
|  |  |  |  |  | Sisp25E03 |
|  |  |  |  |  | Sisp25E05 |
|  |  |  | 25F | Vegetable | Tapu25F01 |
|  | 1400 |  | 25G | Forest |  |
|  |  |  | 25H | Grass |  |
| E86.98,N47.90 | 525.5 | Burqin County, Altay | 26A | Orchard | Puli26A01 |
| E88.05,N47.3 | 551.1 | Beitun City, Altay | 27A | Vegetable | Tapu27A01 |
|  |  |  |  |  | Tapu27A02 |
|  |  |  |  |  | Puji27A03 |
|  |  |  | 27B | Forest | Tapu27B01 |
|  |  |  | 27C | Grass |  |
|  |  |  | 27D | Orchard | Sisp27D01 |
|  |  |  |  |  | Tapu27D02 |
|  |  |  |  |  | Sisp27D03 |
| E89.59,N46.92 | 844.2 | Fuyun County, Altay | 28A | Vegetable | Mero28A01 |
|  |  |  |  |  | Sisp28A02 |
|  |  |  | 28B | Vegetable | Tapi28B01 |
|  |  |  | 28C | Desert |  |
|  |  |  | 28D | Vegetable | Tapu28D01 |
|  |  |  | 28E | Corn |  |
| E87.97,N44.12 | 641.7 | Fukang City, Changji | 29A | Forest | Puli29A02 |
|  |  |  |  |  | Asfu29A03 |
|  |  |  | 29B | Orchard | Asma29B01 |
|  |  |  |  |  | Saki29B02 |
| E89.13,N42.99 | 106.2 | Gaochang, Turpan | 30A | Desert |  |
|  |  |  | 30B | Orchard |  |
|  | -8.24 | Toksun County, Turpan | 30C | Forest |  |
|  |  |  | 30D | Desert |  |
|  |  |  | 30E | Orchard |  |
|  |  |  | 30F | Forest | Asfa30F02 |
|  |  |  |  |  | Puji30F03 |
| E86.31,N42.75 | 1761.2 | Hejing County, Bayingolin | 31A | Desert |  |
|  |  |  | 31B | Forest |  |
|  |  |  | 31C | Grass |  |
|  |  |  | 31D | Vegetable |  |
| E85.78,N43.11 | 3089.4 | Hejing County, Bayingolin | 32A | Desert |  |
|  |  |  | 32B | Desert |  |
|  |  |  | 32C | Forest | Tapu32C01 |
| E86.06,N44.31 | 451 | Farm, Shihezi University | 33A | Corn |  |
|  |  |  | 33B | Vegetable | Tapu33B01 |
|  |  |  | 33C | Cotton | Tapu33C01 |
|  |  |  | 33D | Grass | Tapi33D01 |
|  |  |  | 33E | Cotton | Tapi33E01 |
|  |  |  | 33F | Orchard |  |
|  |  |  | 33G | Cotton |  |
|  |  |  | 33H | Cotton |  |
|  |  |  | 33I | Cotton |  |
|  |  |  | 33J | Cotton |  |
|  |  |  | 33K | Cotton |  |

Table S2 Standard strain information of phylogenetic trees (ITS)

| Species/Strain | ITS GenBank Number |
| --- | --- |
| *Stachybotrys chartarum* CBS 182.80 | NR_145083 |
| *Sarocladium kiliense* MUCL 9724 | NR_130684 |
| *Clonostachys rosea* CBS 154.27 | MH854911 |
| *Metarhizium robertsii* ARSEF 2575 | NR_132011 |
| *Metarhizium flavoviride* ARSEF 2133 | NR_131992 |
| *Chaetomium madrasense* CBS 315.74 | NR_144834 |
| *Cephalotrichum cylindricum* UAMH 1348 | NR_146264 |
| *Paecilomyces maximus*CBS 990.73B | NR_149329 |
| *Penicillium raperi* NRRL 2674 | NR_121230 |
| *Penicillium sizovae* CBS 413.69 | MH859338 |
| *Penicillium citrinum* NRRL 1841 | NR_121224 |
| *Aspergillus fasciculatus* CBS 110.55 | NR_138285 |
| *Aspergillus magaliesburgensis* PPRI 6165 | NR_171610 |
| *Aspergillus insuetus* CBS 107.25 | MH854799 |
| *Aspergillus subramanianii* CBS 138230 | MT955639 |
| *Aspergillus fumigatus* CBS 542.75 | MH860951 |
| *Cordyceps javanica*CBS 134.22 | NR_111172 |
| *Beauveria bassiana*ARSEF 1564 | NR_111594 |
| *Purpureocillium jiangxiense* GDMCC 3.1070 | PP555637 |
| *Purpureocillium lilacinus* CBS 284.36 | MH855800 |
| *Purpureocillium roseum* IOM 325363.1 | MT560195 |
| *Simplicillium spumae* JCM 39053 | LC496916 |
| *Simplicillium obclavatum* CBS 311.74 | MH860859 |
| *Talaromyces purpureogenus* CBS 286.36 | NR_121529 |
| *Talaromyces flavus* CBS 310.38 | NR_147413 |
| *Talaromyces funiculosus* CBS 272.86 | MH_861957 |
| *Talaromyces pinophilus* CBS 631.66 | JN_899382 |
| *Talaromyces wortmannii* CBS 391.48 | MH_856412 |
| *Trichocoma paradoxa* CBS 788.83 | JN899398 |

Table S3 ITS, BenA and CaM Sequences of Tapu14C02 strain

| Gene | Sequence |
| --- | --- |
| ITS | CCCTCGCGGCCCAACCTCCCACCCTTGTCTCCAACACCTGTTGCTTCGGCGGGCCCACCGGGGCCACCCGGTCGCCGGGGGACATCCGTCCCCGGGCCCGCGCCCGCCGAGGCGCTCTGTGAACCCTGATGAAGATGGGCTGTCTGAGTGATATGAAAATTGTCAAAACTTTCAACAATGGATCTCTTGGTTCCGGCATCGATGAAGAACGCAGCGAAATGCGATAAGTAATGTGAATTGCAGAATTCCGTGAATCATCGAATCTTTGAACGCACATTGCGCCCCCTGGCATTCCGGGGGGCATGCCTGTCCGAGCGTCATTTCTGCCCTCAAGCACGGCTTGTGTGTTGGGTGTGGTCCCCCTGGGGACCTGCCCGAAAGGCAGCGGCGACGTCCGTCTGGTCCTCGAGCGTATGGGGCTCTGTCACTCGCTCGGGAAGGACCTGCGGGGGTTGGTCACCACCACATCTTTTTACAAGGTTGACCTCGGATCAGGTAGGAGTTACCCGCTGAACTTAAGCATATCA |
| BenA | TGACCCTTGGCCCAGTTGTTACCAGCACCGGACTGACCGAAAACAAAGTTGTCGGGACGGAAGAGCTGACCAAAGGGACCAGCGCGGACGGCATCCATGGTGCCGGGTTCCAAGTCGACGAGGACAGCACGAGGAACATATTTGTTGCCGGAAGCCTGTTAAGCATTGGATATGAGTTTTTGTTTTTGTTTCTATTGGTTGGTTGTTCGACGCACCTCGTTGAAGTAAACGTTCATACGCTCCAACTGGAGGTCGGAGGAGCCATTGTAACTGTTGATTATCAGATACGGTCGAATTGTAGATGGATTTCGAATCCCATCAACACTTACACGCCGGATCCATCGAGACCGTGCTCAGCAGAGATGATTTGCCTGAAAATAGTCAGCGAGTCGTCGCGACAATTGACTGAAAGCGTGGTCATTCCTCACCAGAAAGCAG |
| CaM | ACGAACTCGTTGTCTAGAAACACAATCACTAATTAGAACAGCTTCGTCCCACATTCTGAATCATATAGGAGGAAACTCACAGTCAATCCTTCCATCACCATCCTGATCAGCCTCACGAATCATCTCATCAACCTCGTCATCGGTCAACTTCTCGCCAATCGAAGTCATGACGTGACGCAATTCAGCTGCAGAGATGAATCCATTGTTGTCACGGTCAAACACCTTGAAAGCCTCACGGATCTCTTCCTCGGAGTCGGTATCCTTCATTTTGCGGGCCATCATTGTCAAGAATTCTAACCATTATCGATCAGTTACCTATCCACAGTAGATTGTAGCGAGAGTCATCATACCAGGGAAATCGATTGTGCCGTTGTTGTCAGCGTCAACTTCGTTGATCATGTCCTGCAATTCGGATTCGGAGGGGTTCTGGCCGAGGGAGCGCATGACGGTGCCCAGTTCCTTGGTTGTGATTTGACCTAATCAATATAGTAAATATCCTGTTCAGAGTCCTATCGTTGATTGCTGCGTGTTCGGGTGAACTCACCATCACCATCCTTGTCCTACTCGTCCAAACGGGCTAGTTAGATAACCGACCAACCAACATACCCAAACAATGATTGCAGGCAGATAGAACTTACGAAAAGGGAGAAAGCCTCCTTGTACTCGGAGACTT |


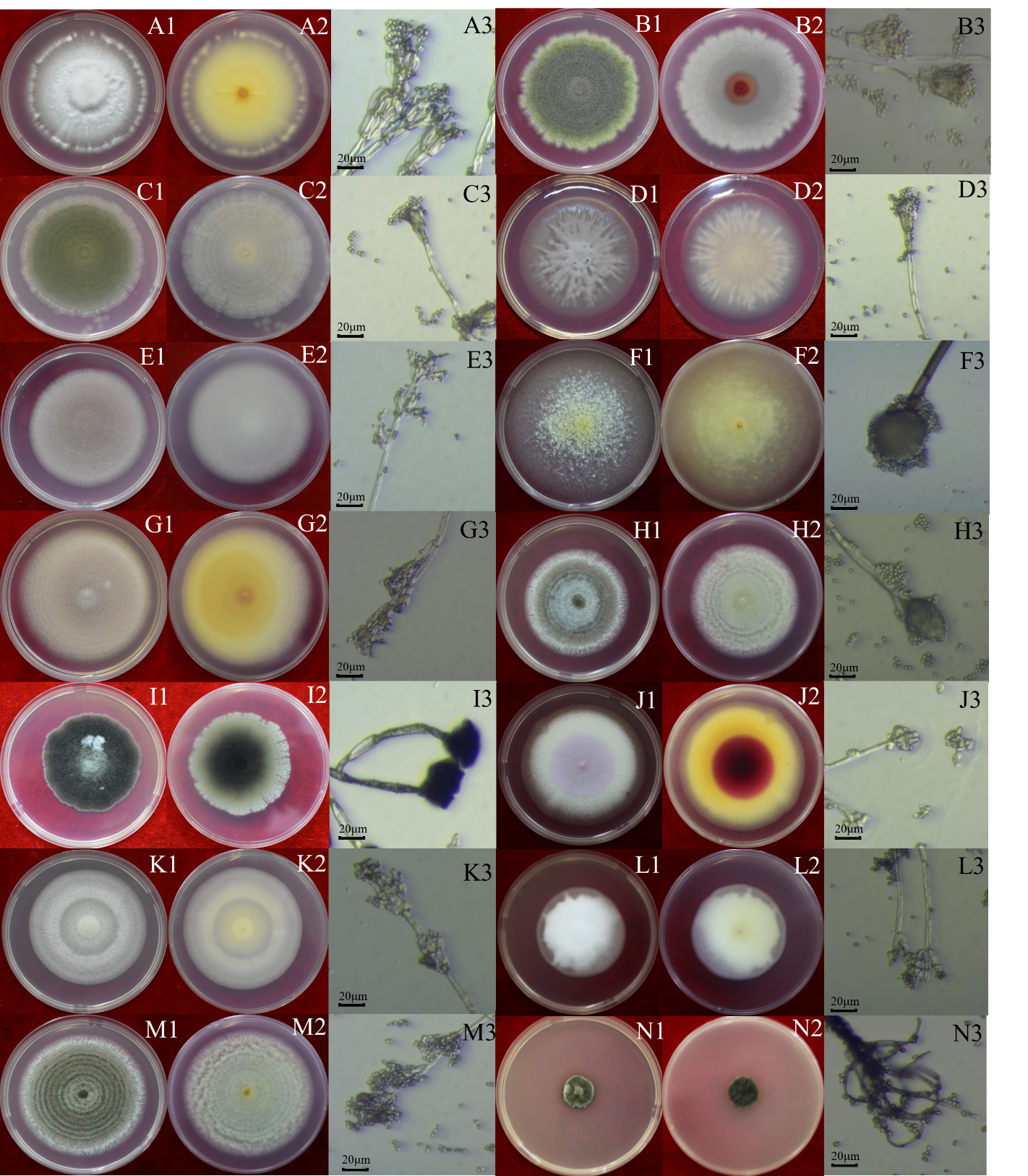


**Fig S1.** Morphological characteristics of the main isolated strains

**1,** indicates the front of a single colony on a PDA plate of isolated fungal strains. **2,** indicates the back. **3,** indicates the morphological structure of the strain under the microscope. **A1-A3,** *S. spumae*; **B1-B3;** *T. funiculosus*; **C1-C3,** *T. purpureogenus*; **D1-D3,** *T. pinophilus*; **E1-E3,** *P. lilacinum*; **F1-F3,** *T. wortmannii*; **G1-G3,** *P. jiangxiense*; **H1-H3,** *As. fumigatus*; **I1-I3,** *S. chartarum*; **J1-J3,** *P. raperi*; **K1-K3**, *C. rosea;* **L1-L3,** *P. citrinum;* **M1-M3,** *M. flavoviride*; **N1-N3,** *C. cylindricum*
